# Sequence-dependent synergistic cytotoxicity of icotinib and pemetrexed in human lung cancer cell lines in vitro and in vivo

**DOI:** 10.1186/s13046-019-1133-z

**Published:** 2019-04-05

**Authors:** Tianze Liu, Lizi Jin, Wenjing Lu, Hairun Gan, Zhidong Lin, Miao Chen, Jiani Liu, Fan Zhang, Siyang Wang, Hongyu Zhang, Wuguo Deng, Hongtao Chen

**Affiliations:** 1 Department of Oncology, The Fifth Affiliated Hospital of Sun Yat-sen University, Zhuhai, 519000 China; 2 State Key Laboratory of Oncology in South China, Sun Yat-sen University Cancer Center, Guangzhou, 510060 China; 3 Guangdong Provincial Engineering Research Center of Molecular Imaging, The Fifth Affiliated Hospital, Sun Yat-sen University, Zhuhai, 519000 Guangdong Province China; 4 Center for Interventional Medicine, The Fifth Affiliated Hospital, Sun Yat-sen University, Zhuhai, 519000 Guangdong Province China; 5 Department of Cardiology, The Fifth Affiliated Hospital of Sun Yat-sen University, Zhuhai, China; 6 Department of General Surgery, The Fifth Affiliated Hospital of Sun Yat-Sen University, Zhuhai, China; 7 Department of Radiation Oncology, The Fifth Affiliated Hospital of Sun Yat-sen University, Zhuhai, China; 8 Department of Clinical Laboratory, The Fifth Affiliated Hospital of Sun Yat-sen University, Zhuhai, 519000 China

**Keywords:** Icotinib, Lung cancer, EGFR mutation, Synergy, Washout period

## Abstract

**Background:**

Recent Clinical trials of administration of epidermal growth factor receptor tyrosine kinase inhibitors (EGFR-TKIs) in combination with standard first-line chemotherapy have failed to improve survival in patients with advanced NSCLC, However, the sequential treatment with EGFR-TKIs and chemotherapy is expected to improve survival of NSCLC. The aim of this study is to test the antiproliferative effect of pemetrexed combined with icotinib in different sequences on non-small cell lung cancer (NSCLC) cell lines to determine the optimal combination schedule, and subsequently elaborated the potential mechanisms.

**Methods:**

Six human lung cancer cell lines with wild-type or mutant EGFR gene were exposed to pemetrexed and icotinib combined in different sequences. Cell proliferation was examined by cell counting kit-8 (CCK-8) and colony formation assay; cell cycle and apoptosis were evaluated by flow cytometry; cell migration and invasion were measured by wound healing and transwell invasion assays respectively; protein expression was by detected by Western blot.

**Results:**

The growth inhibition effect of pemetrexed combined with icotinib on NSCLC cells were schedule-dependent in vitro *and* in vivo. Treatment with pemetrexed followed by icotinib (P-I) had significantly stronger anticancer ability than treatment with icotinib followed by pemetrexed (I-P) and concomitant treatment with pemetrexed and icotinib (P + I). Cell cycle analysis revealed that pemetrexed blocked cells in S phase, whereas icotinib arrested cells in G1 phase. We also found that icotinib markedly enhanced the pro-apoptotic activity of pemetrexed via cytochrome-C/Caspase/Bcl-2 signaling pathway. In addition, our results showed that pemetrexed alone increased the levels of p-EGFR, p-AKT and p-MAPK, which were inhibited by icotinib. Finally, we showed that the washout period of icotinib was no less than 96 h.

**Conclusions:**

Sequential treatment of NSCLC cells with pemetrexed followed by icotinib had powerful antiproliferative effect, and it could become a novel effective combination therapy for NSCLC patients.

## Background

Primary lung cancer is the most common form of cancer in terms of both incidence and death worldwide [1]. Non-small-cell lung cancer (NSCLC) is the most common type of lung cancer and accounts for about 80% of all lung cancer [2], The overall 5-year survival rate for stage IIIB/IV NSCLC is 1–5%, and approximately 70% of NSCLC patients are diagnosed at an advanced stage with local metastasis [3]. Systemic therapy is the backbone of treatments of advanced NSCLC. First-line platinum-based doublet chemotherapy or teratment with epidermal growth factor receptor tyrosine kinase inhibitors (EGFR-TKIs) is optional according to EGFR status [4–9]. However, the benefits of first-line chemotherapy seem to have reached a plateau and only progress free survival (PFS) benefits from EGFR-TKIs. Morevoer, progression of cancer is inevitable even though the standard treatment is given, while second-line treatments such as pemetrexed, docetaxel and EGFR-TKIs, which result in equivalent benefits have a response rate below 10% [6, 10]. It remains an important issue whether EGFR-TKIs and cytotoxic chemotherapy in combination can bring more benefits. Unfortunately, 4 large, randomized phase III clinical trials (INTACT-1, INTACT-2, TALENT and TRIBUTE) of administration of erlotinib or gefitinib in combination with standard first-line chemotherapy have failed to improve survival in patients with advanced NSCLC [11–14]. The failures to achieve the expected positive results could owe to the lack of predictive markers of response to EGFR-TKIs in combination with chemotherapy, or the sequence dependency of the antiproliferative effects of the combination therapies. Therefore, more preclinical experiments are needed to elucidate the mechanism of chemotherapies used in combiantion with EGFR-TKIs in tumor cells to guide rational use of combination therapies in clinical practice.

Pemetrexed is a novel antifolate, which inhibits dihydrofolate reductase through blocking three important metabolic enzymes involved in DNA synthesis: dihydrofolate reductasem (DHFR), glycinamide ribonucleotide formyltransferase, and the most important target-thymidylate synthase [15]. As a first-line therapy for advanced NSCLC, pemetrexed alone has yielded an overall survival (OS) of 4.7 months, and a median progression-free survival (PFS) of 3.3 months [16]. Pemetrexed-based chemotherapy (PBC) has yielded an average OS of 10.3 months [17]. As a single agent in second-line treatment for advanced NSCLC, pemetrexed has yielded a median survival time of 8.3 months and a median PFS of 2.9 months. Also, for maintenance therapy of NSCLC, pemetrexed significantly improved PFS from 2.6 months to 4.3 months [18]. Because of the exact curative effect, pemetrexed was approved for NSCLC in 2008 by Food and Drug Administration (FDA).

Icotinib hydrochloride, similar to gefitinib and erlotinib, is a potent EGFR-TKI. In vitro preclinical studies reported that icotinib selectively inhibited the EGFR members including both wild-type and mutants with inhibition efficacies of 61–99%, without affecting the other 81 kinds of kinases [19, 20]. The phase III trial (ICOGEN) with a randomized, double-blind, multicenter, controlled, head-to-head study design indicated that the efficacy differences were not significant between the icotinib-treated group and the gefitinib-treated group [21]. The objective response rate (ORR) of the icotinib group was 27.6% versus 27.2% of the gefitinib group, and the disease control rate (DCR) of the icotinib group was 75.4% versus 74.9% of the gefitinib group. The PFS in the icotinib group was 4.6 months versus 3.4 months in the gefitinib group. ICOGEN also demonstrated the safety and efficacy of icotinib for advanced NSCLC patients for whom platinum-based chemotherapy had failed. Due to its positive anti-tumor activities in advanced NSCLC patients, especially in those with EGFR mutations, icotinib has recently been approved by the State Food and Drug Administration of China.

Here, we investigated the combinatorial effect generated by sequential application of pemetrexed and icotinib, and identified the underlying mechanism of actions in human NSCLC cell lines.

## Materials and methods

### Drugs and reagents

Icotinib (99.9% purity) was graciously supplied by Zhejiang Beta PharmaInc (Zhejiang, China), and dissolved in dimethyl sulfoxide (DMSO) to 50 mM for stock solution. Pemetrexed was kindly provided by Haosen pharmaceutical company (Jiangsu, China), and dissolved in 0.9% NaCl to a final concentration of 21.2 mM for stock solution. Both drugs were stored at − 20 °C and diluted with culture medium before use. Anti-EGFR, anti-pEGFR, anti-AKT, anti-pAKT, anti-MAPK, and anti-pMAPK antibodies were purchased from Cell Signaling Technology (Danvers, MA). Anti-cyclin A and anti-cyclin E antibodies were were obtained from Bioworld Technology (St Louis Park, MN). Cell counting kit-8 was purchased from Dojindo Laboratories (Kumamoto, Japan).

### Cell lines

Human NSCLC cell lines A549, H1975 and PC-9, HCC827, H1299, and H460 were obtained from the American Type Culture Collection (ATCC, Manassas, VA). All cell lines were grown in RPMI 1640 supplemented with 10% fetal bovine serum (FBS), penicillin (100 U/ml) and streptomycin (100 μg/ml) at 37 °C in a humidified atmosphere with 5% CO2. A549H, 1299, and H460 cells express wild-type EGFR. PC-9 and HCC827 cells express mutant type EGFR, whereas H1975 cells carry the EGFR L858R-T790 M mutation and are resistant to EGFR-TKI.

### CCK-8 cell proliferation assay

Cells were seeded in 96-well plates (2000 cells per well) and exposed to serial dilutions of icotinib, pemetrexed for 72 h. Cell viability was assayed by CCK-8. Growth inhibition was depicted as the percentage of surviving drug-treated cells versus PBS-treated control cells. The IC50 value was the concentration leading to 50% cell growth inhibition compared with untreated control. To evaluate the antiproliferative effects of the combined treatment, cells were treated in three different sequences: 1) pemetrexed for 24 h, old medium removed and followed by icotinib for 48 h (P-I); 2) icotinib for 48 h, old medium removed and followed by pemetrexed for 24 h (I-P); 3) treated concomitantly with pemetrexed and icotinib for 72 h (P + I). Interactions between icotinib and pemetrexed were evaluated by the CalcuSyn software and presented as the combination index (CI): CI < 0.9 represents synergistic effect; 0.9 ≤ CI ≤ 1.1 represents additive cytotoxicity; and > 1.1 represents antagonistic effect.

### Colony formation assay

Cells were cultured and treated with pemetrexed and/or icotinib in the indicated concentrations and sequences for 72 h before they were plated in 6-well plates (500 single cells per well). After incubation for another 12 days, cells were washed twice with phosphate buffered saline (PBS), fixed with paraformaldehyde for 15 min, and stained with crystal violet for colony counting. All assays were independently performed in triplicates.

### Wound healing assay

Approximately 1 × 10^5^ PC-9 or A549 cells were seeded in each well of 6-well plates. After overnight incubation, cells were either untreated or treated with pemetrexed and icotinib in the indicated concentrations and sequences. When cell confluence reached about 90–100% after treatment, wounds were created in confluent cells using a 200 μL pipette tip. The cells were rinsed several times with PBS to remove any freely floating cells and debris, and cultured in growth medium. Wound healing was observed at different time points (0, 24, 48 h), and the wound gap was photographed and measured.

### Transwell invasion assay

Matrigel invasion assays were performed using a 24-well cell culture insert and BD Matrigel (BD Biocoat, Bedford, MA). The transwell membrane was coated with diluted Matrigel (30 μL). The lower chambers were filled with 500 μL of RPMI 1640 medium containing 20% fetal bovine serum. The cell suspension in FBS-free medium (5 × 10^4^ cells) was added to the upper chamber and incubated for 24 h. The cells that had not migrated through the pores were manually removed from the upper face of the filters using cotton swabs, and cells adherent to the bottom surface of the inserts were fixed in cold paraformaldehyde for 15 min and stained with crystal violet. Finally, the filters were washed thoroughly in water and images were taken under a microscope with appropriate magnification. These experiments were done in triplicates.

### Cell cycle analysis

Cells were seeded into six-well plates at a density of 1 × 10^5^ cells per well. After 48 h, the wells were treated with pemetrexed and icotinib, respectively. At the end of the experiments, adherent cells were trypsinized, counted, washed, and resuspended, along with the corresponding floating cells. The cells were then pelleted and fixed by dropwise addition of 70% ice-cold ethanol at 4 °C overnight. The fixed cells were washed with PBS and stained with 50 μg/mL propidium iodide, 50 μg/mL RNase I and 0.2% Triton X-100 in the dark at 37 °C for 30 min and then analyzed with flow cytometry.

### Cell apoptosis analysis

Apoptosis was measured by flow cytometry using Annexin V-FITC and PI double staining method. According to the manufacturer’s protocol, cells were seeded into six-well plates at a density of 1 × 10^5^ cells per well, treated with pemetrexed or icotinib at double IC50 levels for 72 h. Cells were then collected and resuspended in 500 μL of binding buffer containing 5 μL of Annexin V-fluorescein isothiocyanate and 5 μL of propidium iodide, and then incubated for 15~30 min in the dark at room temperature and analyzed using flow cytometry.

### Western blot analysis

Cell lysates were prepared and the procedures for western blot were done as follows: equal amounts of protein (30 μg) from each sample were resolved by 6, 10% or 12% sodium dodecyl sulfate-polyacrylamide gel electrophoresis (SDS-PAGE) and transferred to a PVDF membrane. The blots were probed with specific antibodies and the protein signals were visualized by an enhanced chemiluminescence reaction system, as recommended by the manufacturer. Equal loading was assessed by immunoblotting of the amount of GAPDH.

### Confocal immunofluorescence

PC-9 cells grown on chamber slides were washed with PBS, fixed with paraformaldehyde and permeabilized with pre-cooled methyl alcohol for 10 min at − 20 °C. The samples were then pretreated with 10% bovine serum albumin (BSA) in PBS, and then specific antibodies were added in and incubated at 4 °C overnight. After washing the samples with PBS for 5 min for three times, secondary antibodies were added in and incubated for 1 h. After five additional 5-min washes, samples were examined using the confocal microscope.

### Animal study

Six-week old BALB/c nude mice from the Beijing Vital River Laboratory Animal Technology Co., Ltd. (Beijing, China) were maintained in a specific pathogen-free environment. All the operations were carried out according to the Guide for the Care and Use of Laboratory Animals (NIH publications Nos. 80–23, revised 1996) and the Institutional Ethical Guidelines for Animal Experiments developed by Sun Yat-sen University. PC-9 cells (5 × 106 in 100 μL PBS) were injected subcutaneously into the left flank of each mouse. When the formed tumor reached 0.1 cm3 after cell inoculation, the animals were randomly divided into six groups with five mice in each group. The first group of mice were intratumorally injected with PBS as control (C), the second received icotinib (60 mg/kg) treatment alone (I), the third received pemetrexed (100 mg/kg) treatment alone (P), the fourth received icotinib and pemetrexed (P + I), the fifth were treated with pemetrexed followed by icotinib (P-I), and the sixth were treated with icotinib followed by pemetrexed (I-P). The tumor size was measured using vernier calipers once every 2 days, and the tumor volume was calculated as V = (width2 × length)/2. The experiment was terminated 22 days after tumor cell inoculation, and the mice were sacrifced. The tumors from each mouse were excised, weighed, embedded in paraffin and sectioned.

### Immunohistochemistry staining

Xenograft tumor tissues were analyzed by immunohistochemistry staining using special antibodies. Briefly, after deparaffinizing, blocking and antigen retrieval, the tumor sections were incubated with primary antibodies overnight at 4 °C. After washing, tumor sections were incubated at room temperature with horseradish peroxidase-conjugated anti-goat antibodies for 30 min, and colors were developed with 3,5-diaminobenzidine (DAB) substrate followed by Mayer’s hematoxylin counterstaining.

### Statistical analysis

Results are presented as mean ± SE of at least three experiments. Differences between the mean values of the two subgroups were evaluated using Student’s t-test. Differences between the mean values of three subgroups were compared by one-way analysis of variance (ANOVA). *p* < 0.05 was considered to be a statistically significant in this study. SPSS 13.0 software was used for all statistical analysis.

## Result

### Icotinib-enhanced cytotoxicity of pemetrexed is sequence-dependent regardless of the mutation status of EGFR in vitro

**Fig. 1:**
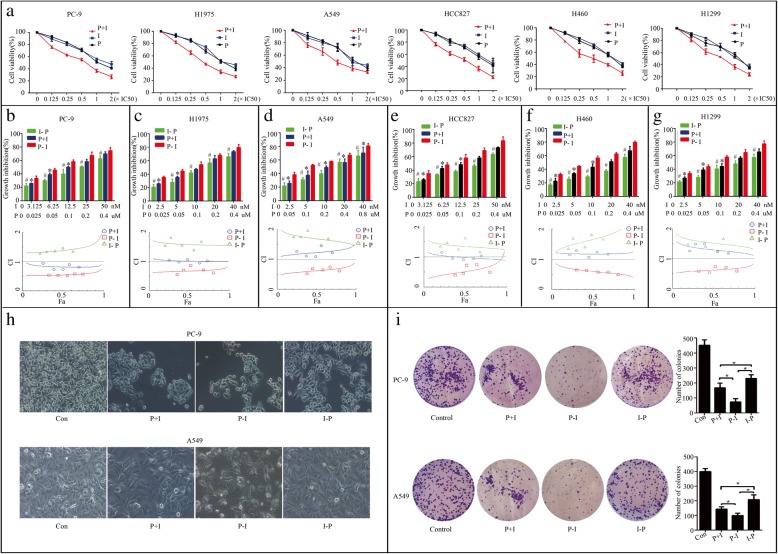
Icotinib enhanced the cytotoxicity of pemetrexed in vitro in a sequence-dependent manner. **a** Six NSCLC cell lines were exposed to pemetrexed and icotinib together or alone using constant ratios of the IC50 dose, respectively. **b**-**g** Cell lines were exposed to different combined models of pemetrexed and icotinib. #*p* < 0.05, I-P versus P-I. *p < 0.05, P + I versus P-I. Combination indexes (CI) between icotinib and pemetrexed were evaluated in six NSCLC cell lines. The CI value: < 0.9 represents synergistic effect, 0.9~1.1 represents addictive cytotoxicity, > 1.1 represents antagonistic effect. **h** The changes in cell morphology and spreading in PC-9 and A549 cells treated with pemetrexed and icotinib with the concentrations of two times of each cell’s IC50 at three sequences were observed. **i** Representative images and quantification of colony formation assay. Data are presented as mean ± SD of triplicate experiments, *p < 0.05

In our study, the antiproliferative effects treated with pemetrexed and icotinib together or alone on six NSCLC cell lines (PC-9, H1975, A549, HCC827, H460, and H1299) were concentration-dependent (Fig. 1a), and the growth inhibition effect of pemetrexed and icotinib together was more potent than that of icotinib or pemetrexed alone in each dose we used. The IC50 values of pemetrexed for PC-9, H1975, A549, HCC827, H1299 and H460 were 0.20 ± 0.05 μM, 0.17 ± 0.03 μM, 0.37 ± 0.10 μM, 0.25 ± 0.08 μM, 0.18 ± 0.08 μM, 0.34 ± 0.07 μM, respectively. The IC50 values of icotinib for PC-9, H1975, A549, HCC827, H1299 and H460 were 26.80 ± 3.62 nM, 18.80 ± 0.40 μM, 21.8 ± 0.60 μM, 24.40 ± 2.88 nM, 19.50 ± 2.86 uM, and 23.60 ± 0.30 μM, respectively. The result showed PC-9 and HCC827 cells were much more sensitive to icotinib than A549, H1975, H1299 and H460 cells. We then evaluated the growth inhibition effect on PC-9, A549, and H1975 of the combination treatment with pemetrexed and icotinib in three different sequences. We used 0.125, 0.25, 0.5, 1 and 2 times the IC50 dose of pemetrexed and icotinib, respectively. The result showed that the growth inhibition effect of pemetrexed followed by icotinib (P-I) was more potent than that of icotinib followed by pemetrexed (I-P) in each dose we used, and more potent than concomitant administration of the two drugs (P + I) in median and low doses in PC-9, H1975, HCC827, H1299 and H460 cells and in each dose used in A549 cell line (Fig. 1b-g). The CI value showed the antiproliferative effects of the pemetrexed-icotinib (P-I) sequence all resulted in synergy (CI < 1) even in EGFR-TKI-resistant cells. The sequence of icotinib-pemetrexed (I-P) resulted in antagony (CI > 1) even in EGFR-TKI-sensitive cell lines. Concomitant administration of the two drugs (P + I), however, generated synergistic, or additive, or antagonistic effects in six cell lines (Fig. 1b-g). PC-9 cell lines was chosen for further studies as it was sensitive to both icotinib and pemetrexed.

We then analyzed the effect of pemetrexed and icotinib on cell morphology in PC-9 and A549 cells. Treatment with P-I and P + I at two times of each cell’s IC50 reduced cell-to-cell contact more obviously and led to a lower spreading with fewer formation of filopodia by comparison with the I-P group (Fig. 1h). Colony formation is an immensely important parameter in cancer survival and progression, so we next corroborated these effects by performing colony formation assays. As is shown in Fig. 1i, group P-I and P + I inhibited colony formation more markedly than group I-P. Moreover, the number of colonies was significantly decreased in PC-9 cells treated with P-I compared with P + I.

### Icotinib enhanced the inhibition effect of pemetrexed in NSCLC cells growth through EGFR and AKT/ERK signaling pathway

**Fig. 2:**
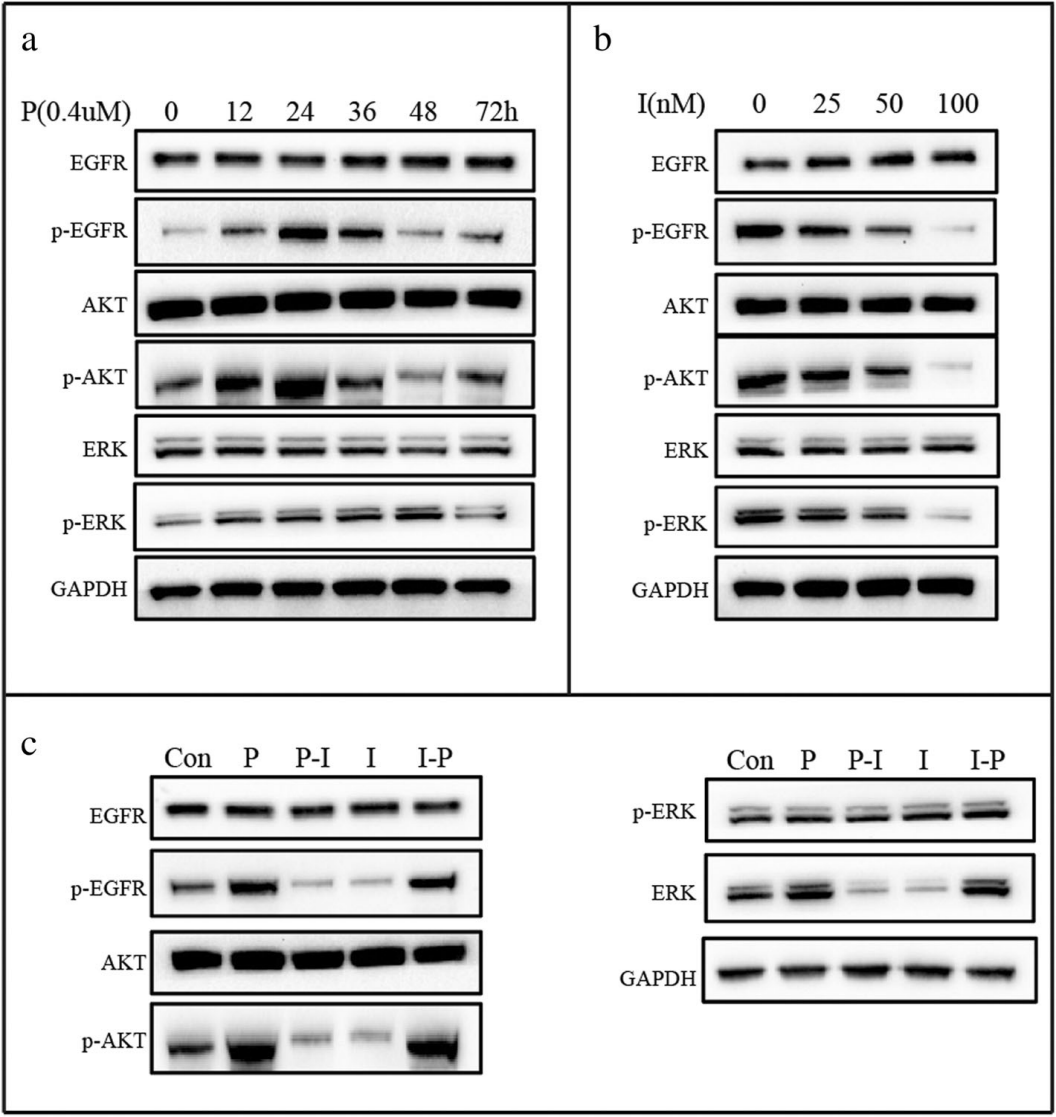
Effect of pemetrexed and icotinib on EGFR and AKT/ERK signaling pathways. After treatment with incremental exposure time of pemetrexed (**a**), incremental concentration levels of icotinib (**b**) and sequential application of pemetrexed and icotinib (**c**), the total and phosphorylated EGFR, AKT, and MAPK proteins in PC-9 cells were detected by Western blot. GAPDH served as the loading control. The gray value below protein bands were quantified by Image J software

To gain insight into the molecular mechanisms of the schedule-dependent synergistic interaction between pemetrexed and icotinib, we detected the effect of drugs on EGFR and AKT/ERK signaling pathway. Firstly, PC-9 cells were treated with increasing exposure time to pemetrexed with the concentration of 0.4 μM. Compared with control groups, we observed the level of p-EGFR increased to the peak at the exposure time of 12 h and last for another 12 h before its decrease. The level of p-AKT increased at the time of 12 h and decreased to the level below the control level after 36 h. The level of p-ERK resulted in an initial increase lasting for 48 h before it dropped down at 72 h (Fig. 2a). Secondly, cells were exposed to increasing concentrations of icotinib. As is shown in Fig. 2b, Compared with control, concentration-dependent down-regulation was found at the p-EGFR, p-AKT, and p-ERK levels. In addition, we tried to uncover the dynamic changes of the expression of p-EGFR, p-AKT, and p-ERK when treated with pemetrexed followed by icotinib (P-I) or icotinib followed by pemetrexed (I-P). Cells were exposed to double IC50-value doses of drugs with pemetrexed followed by icotinib (P-I) or icotinib followed by pemetrexed (I-P). As is shown in Fig. 2c, the levels of p-EGFR, p-AKT, and p-ERK increased markedly when cells exposed to pemetrexed compared with control group. However, the phosphorylation levels of EGFR, AKT, and ERK were reduced by icotinib administered after pemetrexed. Conversely, the expression of p-EGFR, p-AKT, and p-ERK was first down-regulated significantly after icotinib exposure for 48 h, but subsequent pemetrexed exposure for 48 h up-regulated the levels of p-EGFR, p-AKT, and p-ERK. These results suggested that pemetrexed activated the EGFR and its downstream AKT/ERK signaling pathways in favor of the survival of tumor cells while icotinib inactivate these pathways. Therefore, pemetrexed followed by icotinib contributes to synergistic antiproliferative effect. In contrast, in the reverse sequence, icotinib blocked these pathways but pemetrexed reactivated them, which resulted in antagonism.

### Icotinib enhanced the inhibition effect of pemetrexed on cell migration and invasion in a sequence-dependent manner

**Fig. 3:**
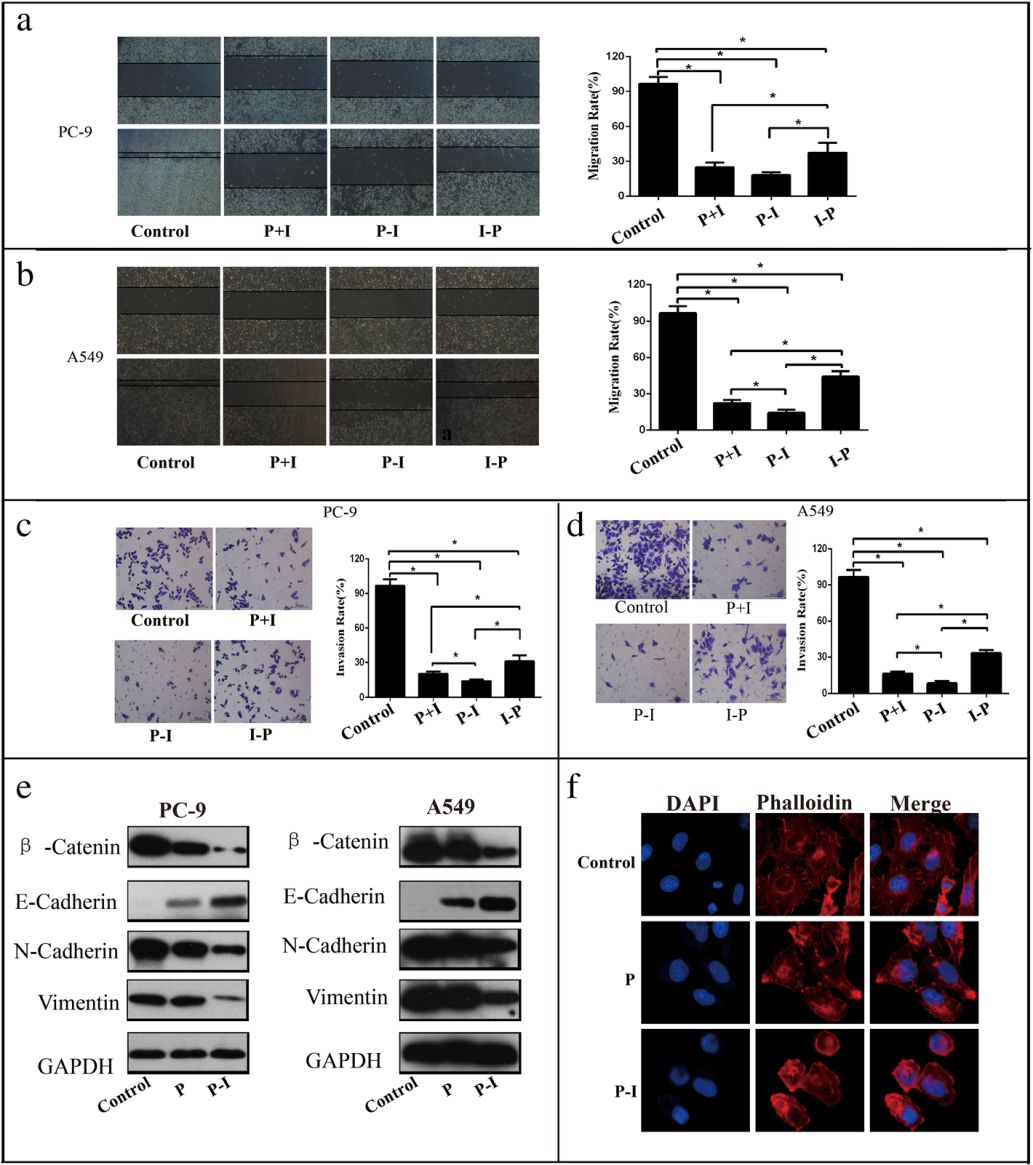
Icotinib enhanced the inhibition effect of pemetrexed in migration and invasion in a sequence-dependent manner. **a**, **b** Representative images and quantification of the results of wound healing assay in PC-9 (**a**) and A549 cells (**b**). The magnification of the microscope is 40×. Data are presented as mean ± SD of triplicate experiments. **c**, **d** Representative images and quantification of the results of transwell invasion assay in PC-9 (**c**) and in A549 cells (**d**). The magnification of the microscope is 100×. **e** The EMT related protein molecules were analyzed by western blot in PC-9 and A549 cells treated with pemetrexed with or without icotinib. **f** Cytoskeleton was analyzed by immunofluorescence assay for phalloidin (red) with DAPI counterstaining (blue) in PC-9 cells treated with pemetrexed with or without icotinib (400×). Data are presented as mean ± SD of triplicate experiments, *p < 0.05

Effects of pemetrexed and icotinib on cell migration were detected by wound healing assay. According to our results, P + I and P-I reduced cell migration more significantly in PC-9 and A549 cells, compared with the I-P group (Fig. 3a and b). Moreover, wound healing assay also demonstrated that A549 cells in group P-I moved significantly slower than group P + I (Fig. 3b). We also performed transwell invasion assay and found that the number of cells migrating was significantly lower when PC-9 and A549 cells were treated with P + I or P-I than with I-P (Fig. 3c and d). No obvious change was found between group P + I and group P-I. Moreover, as epithelial-to-mesenchymal transition (EMT) is an important way for metastasis, we next examined the cytoskeleton by immunofluorescence assay and the expression of proteins related to EMT (β-Catenin, E-cadherin, N-cadherin and vimentin) by western blot. The results showed that the number of spike-like flopodia at the edges of the cells treated with pemetrexed was decreased, and the decrease was more prominent in PC-9 cells treated with pemetrexed followed by icotinib (Fig. 3f). Also, the expressions of the EMT related proteins β-Catenin, N-cadherin, and vimentin were down-regulated while E-cadherin was up-regulated in cells treated by pemetrexed, which were more significant than treatment with pemetrexed followed by icotinib (Fig. 3e).

### Icotinib enhance pemetrexed’s pro-apoptosis effect markedly via cytochrome-C/ caspase/Bcl-2 signaling pathway

**Fig. 4:**
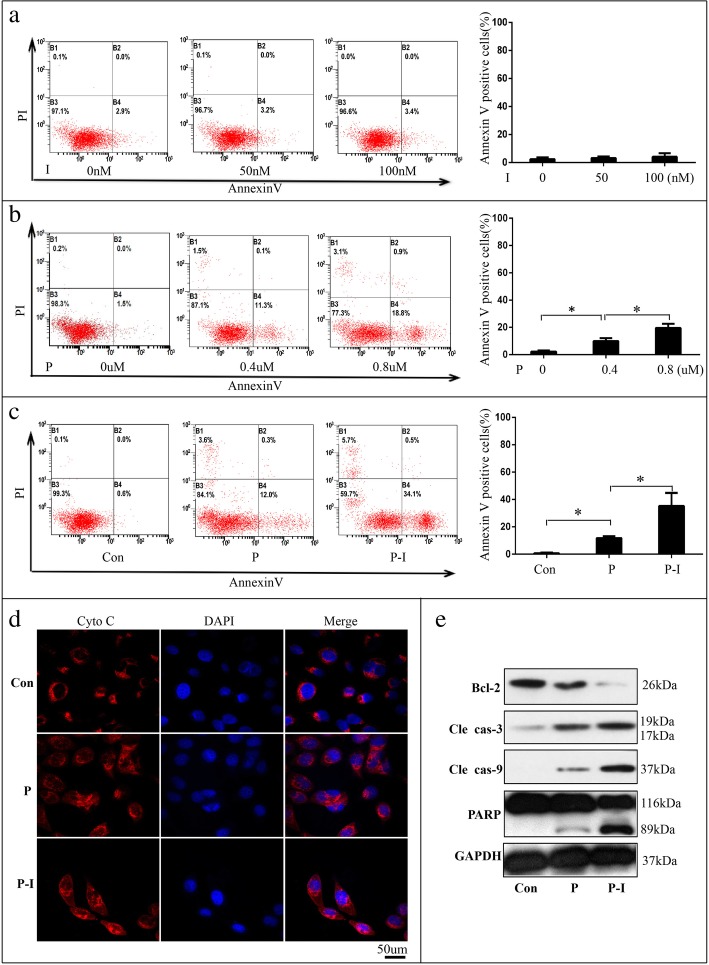
The effect of pemetrexed(P) and icotinib(I) on inducing apoptosis of PC-9 cells in vitro. **a** PC-9 cells were treated with incremental concentration levels of icotinib. **b** PC-9 cells were treated with incremental concentration levels of pemetrexed. **c** PC-9 cells were treated with pemetrexed with or without icotinib. The quantitative analysis was shown in the bar graphs. **d** The release of cytochrome C (cyto-C) was monitored by immunofluorescence analysis in PC-9 cells treated with pemetrexed with or without icotinib (400×). **e** The levels of the bcl-2, cleaved caspase-3, caspase-9 and PARP proteins were analyzed by Western blot in PC-9 cells treated with or without pemetrexed with or without icotinib. The quantitative analysis was shown in the bar graphs. Data are presented as mean ± SD of triplicate experiments, *p < 0.05

Apoptosis is a process of programmed cell death, and most anticancer drugs function primarily by inducing apoptosis [22]. We analyzed the effect of icotinib and pemetrexed on apoptosis. PC-9 cells were seeded into 6-well plates (1 × 10^5^ cells per well) and treated with concentrations of 2 and 4 times of the IC50 of icotinib or pemetrexed for 72 h. The results demonstrated that the percentages of apoptotic cells were elevated markedly after treatment with 0.8 μM pemetrexed (Fig. 4b), but only slightly after treatment with concentrations of 4 times of the IC50 of icotonib (Fig. 4a). Surprisingly, treatment with pemetrexed followed by icotinib induced more apoptotic cells compared with pemetrexed alone (Fig. 4c), which means icotinib could enhance pemetrexed’s pro-apoptosis markedly when treated after pemetrexed though it could not induce apoptosis alone. To elucidate the potential molecular mechanisms, we first performed immunofluorescence to monitor the subcellular localization of cytochrome C (Cyto C), an upstream molecule of the caspase cascade-dependent apoptotic signaling pathway. Figure 4d showed that treatment with pemetrexed triggered the release of cytochrome C from mitochondria to cytoplasm, which was further enhanced in cells with treatment of pemetrexed followed by icotinib. We next analyzed the proteins of caspase and bcl-2, which were involved with apoptosis [23]. We found that the expressions of cleaved PARP, cleaved caspase 3 and cleaved caspase 9 were up-regulated while the expression of bcl-2 was down-regulated, and the expression change of these proteins was more obvious in cells with treatment of pemetrexed followed by icotinib (Fig. 4e). The result revealed that icotinib enhanced pemetrexed’s pro-apoptosis effect via cytochrome-C/caspase/bcl-2 signaling pathway.

### Icotinib enhanced the inhibition effect of pemetrexed by arresting cells in G1-phase

**Fig. 5:**
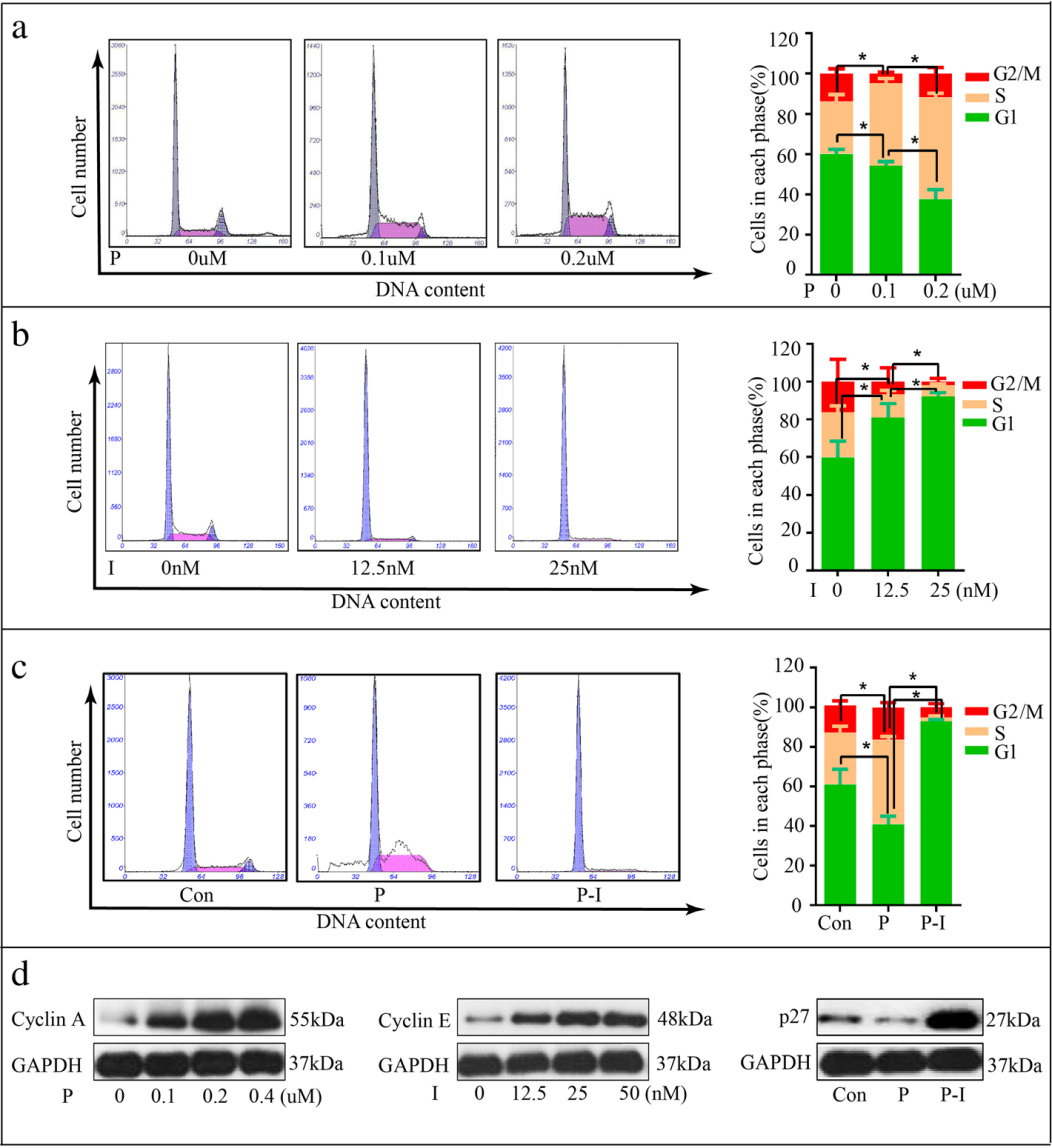
The effect of pemetrexed and icotinib on cell cycle in PC-9 cells. **a** The cell cycle distribution of PC-9 cells with the treatment of pemetrexed. **b** The cell cycle distribution of PC-9 cells with the treatment of icotinib. **c** The cell cycle distribution of PC-9 cells with the treatment of pemetrexed or pemetrexed followed by icotinib. **d** Western blot showed the changes of cyclinA, cyclinE and p27 after treatment with pemetrexed or pemetrexed followed by icotinib. The quantitative analysis was shown in the bar graphs. Data are presented as mean ± SD of triplicate experiments, *p < 0.05

Flow cytometry analysis was performed to evaluate the effect of sequential pemetrexed and icotinib treatments on cell cycle distribution and to find whether their cell cycle modulation activity could provide clues to explain the different combination effects. Our results showed the percentages of the cells in S phase significantly increased with treatment of pemetrexed (Fig. 5a) while cells in the G1 phase increased substantially when exposed to icotinib (Fig. 5b). These suggest that pemetrexed blocks cells in the S phase and icotinib arrests cells in the G1 phase. Also, we found the cells were arrested in the G1 phase with treatment of pemetrexed followed by icotinib (Fig. 5c). To further understand the regulatory mechanism and confirm the effect of the drugs on cell cycle, we detected the expressions of various cell cycle related proteins, finding that the expression of cyclin E was up-regulated after treatment of icotinib, consistent with G1-phase arrest. Pemetrexed increased the expressions of cyclin A, which was consistent with S-phase arrest (Fig. 4e). In addition, p27 was down-regulated by pemetrexed first and up-regulated after treatment with icotinib.

### The washout period of icotinib was no less than 96 h

**Fig. 6:**
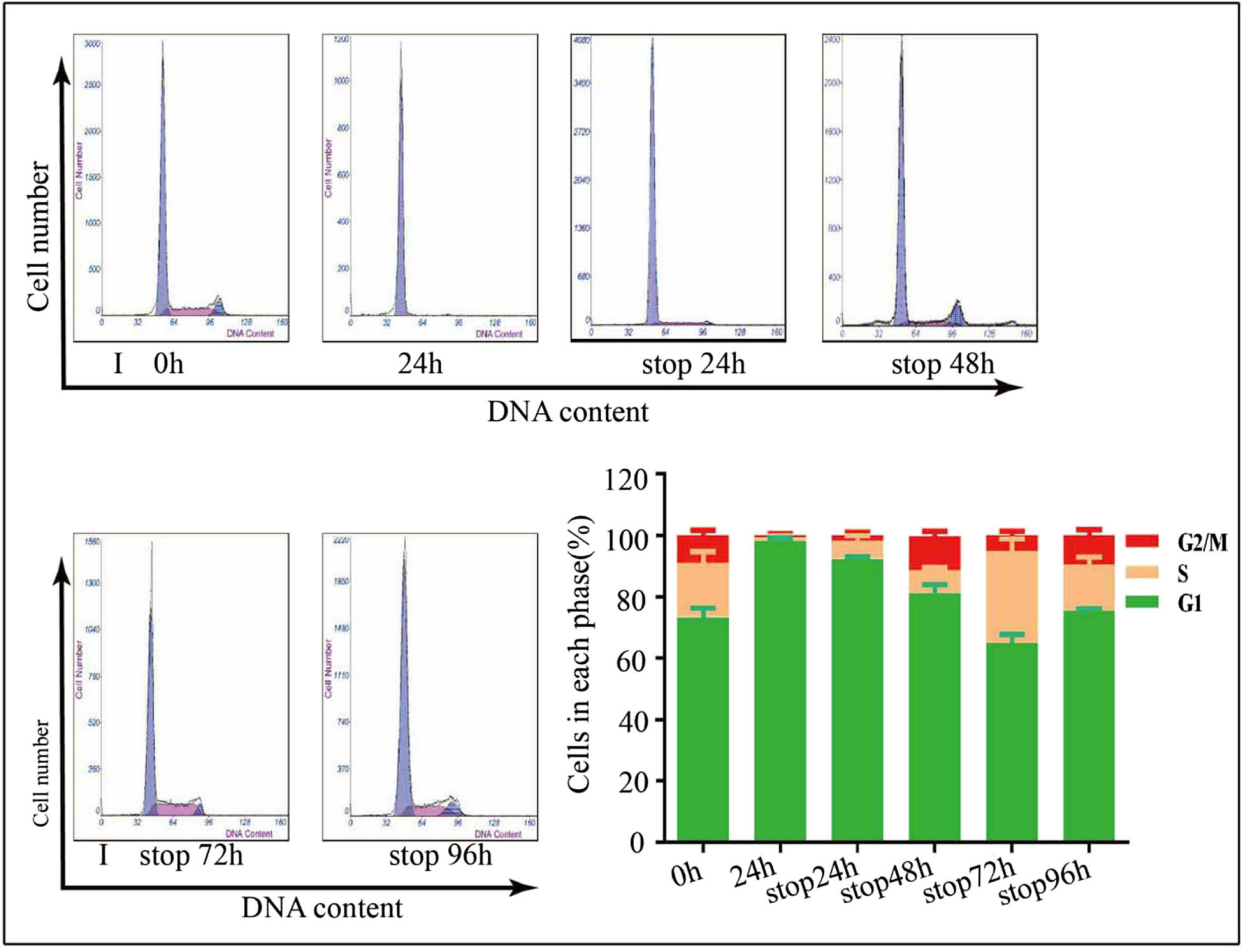
The washout period of icotinib was measured by flow cytometry. PC-9 cells were treated with double IC50 doses with sterile PBS and then red with fresh medium free of icotinib. Cell cycle were exam of icotinib. About 48 h later, medium with icotinib was removed and cells were washed three timeined at 0 h, 24 h, 48 h, 72 h, and 96 h after icotinib was removed. The quantitative analysis was shown in the bar graphs. Data are presented as mean ± SD of triplicate experiments, *p < 0.05

As the tumor therapy is divided into multiple courses, it is important to avoid antagony generated between icotinib in the first course and pemetrexed in the second course. To find out the washout period of icotinib in cell lines, we treated PC-9 cells with double IC50 dose of ciotinib for 48 h, aspirated and washed with PBS three times, and then continue to culture cells with fresh medium free of icotinib. At last, we tested the cell cycle distribution every 24 h after removing medium with icotinib. About 98.2% cells were arrest in the G1 phase when treated with 50 nM icotinib for 48 h, and the percentage of G1 phase cells were 90.2, 85.1, 65.5, 74.75% after 24 h, 48 h, 72 h, and 96 h after changing to fresh medium free of icotinib (Fig. 6). This result showed the distribution of cell cycle modulated by icotinib was eliminated no less than 96 h.

### Icotinib enhanced the cytotoxicity of pemetrexed in vivo in a sequence-dependent manner

**Fig. 7:**
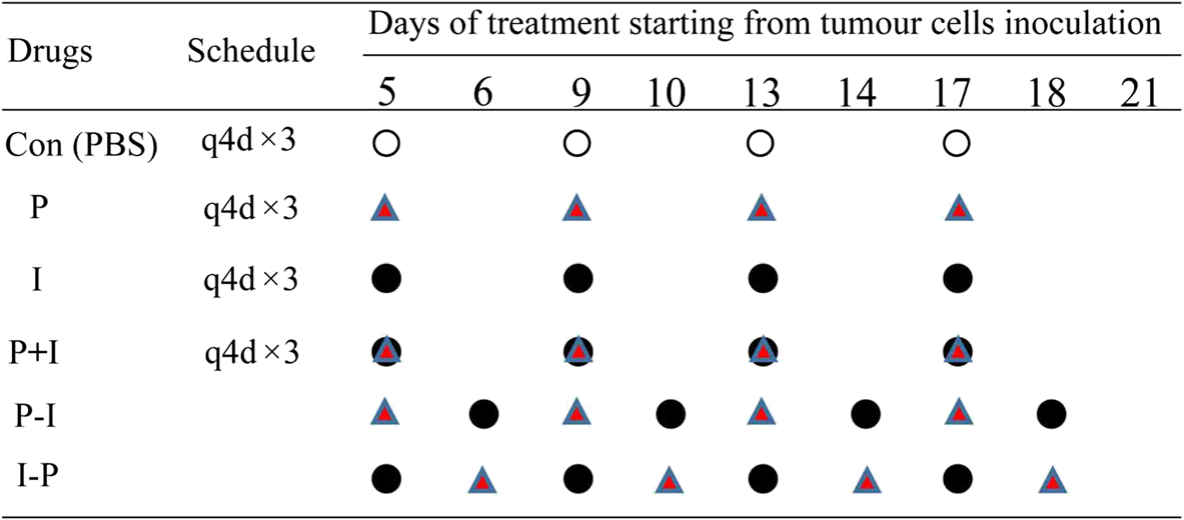
Schematic representation of the treatment schedule and dose for pemetrexed and icotinib in the xenograft study

**Fig. 8:**
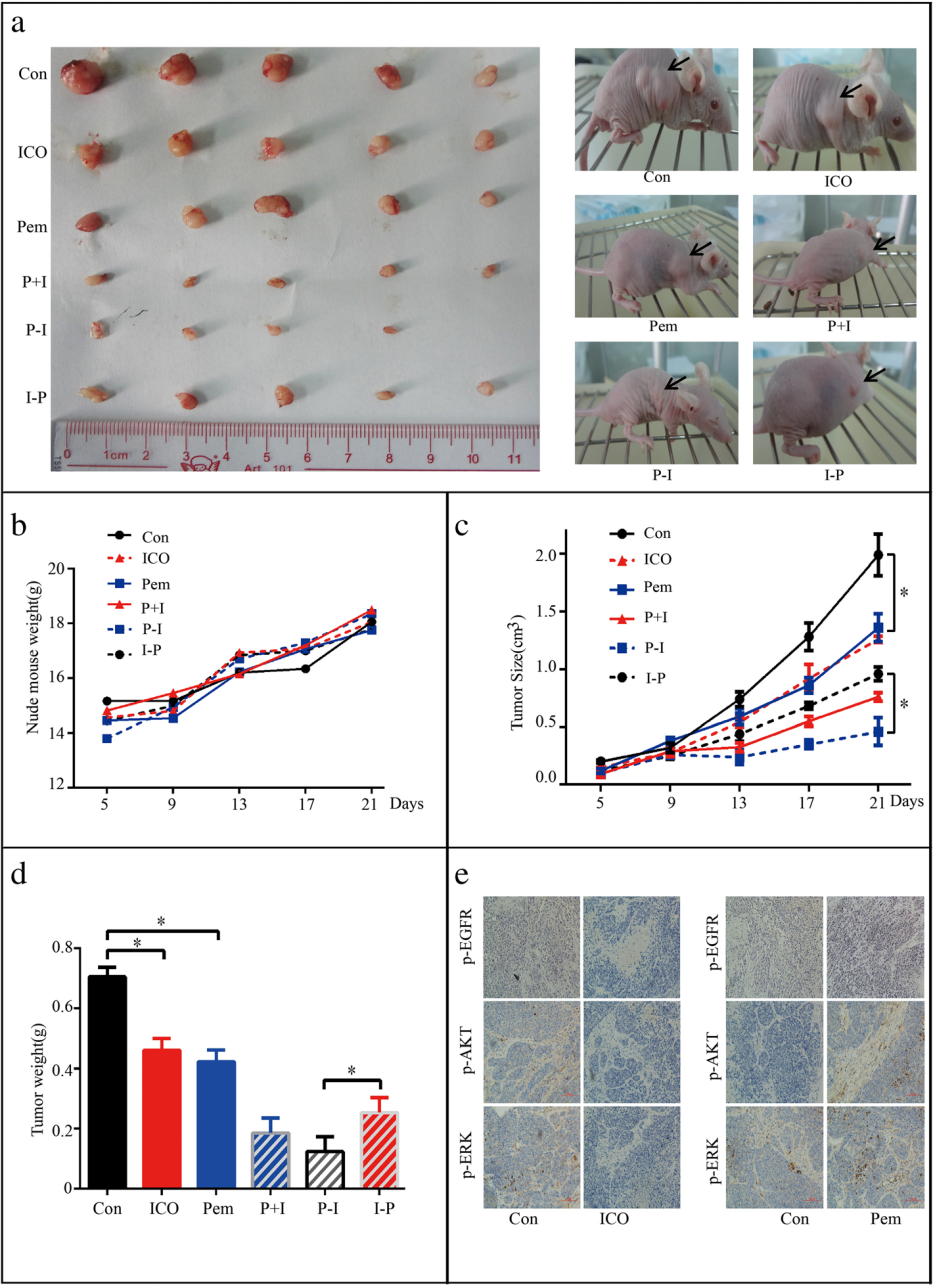
Icotinib enhanced the cytotoxicity of pemetrexed in vivo in a sequence-dependent manner. **a** Tumors from mice after administration of icotinib or pemetrexed alone or the two together in different sequences. **b** Mouse body weights after administration of icotinib or pemetrexed alone or the two together in different sequences. **c** Tumor volume after administration of icotinib or pemetrexed alone or the two together with in sequences. **d** Tumor weight after administration of icotinib or pemetrexed alone or the two together in different sequences. **e** Representative immunohistochemical staining of p-EGFR, p-AKT and p-ERK from tumour sections. Data are presented as mean ± S.E. * *p* < 0.05

Based on the results from in vitro studies, we further investigated whether icotinib enhanced the cytotoxicity of pemetrexed in NSCLC xenograft mouse models. PC-9 cells were injected subcutaneously into the left flank of nude mice, and visible tumors developed at the injection sites after 5 days with mean tumor volume of 0.1 cm^3^. Thirty BALB/c nude mice were randomly divided into six treatment groups (Fig. 7). Afterward, icotinib or pemetrexed alone, or the two together with different sequences were administered for 17 days. As is shown in Fig. 8a-d, the tumor volume and tumor weight were decreased in the group treated with icotinib or pemetrexed alone when compared to the PBS control group, and further decreased in icotinib combined with pemetrexed treatment group. Moreover, we found P-I group showed stronger growth inhibition on xenografts than treatment with I-P. We next analyzed the expression of p-EGFR, p-AKT and p-ERK by immunohistochemical staining. The results showed the expressions of p-EGFR, p-AKT and p-ERK were down-regulated by icotinib but was up-regulated by pemetrexed (Fig. 8e). These data further indicated that icotinib enhanced the cytotoxicity of pemetrexed in NSCLC via EGFR/AKT/ERK signaling pathway in vivo.

## Discussion

**Fig. 9:**
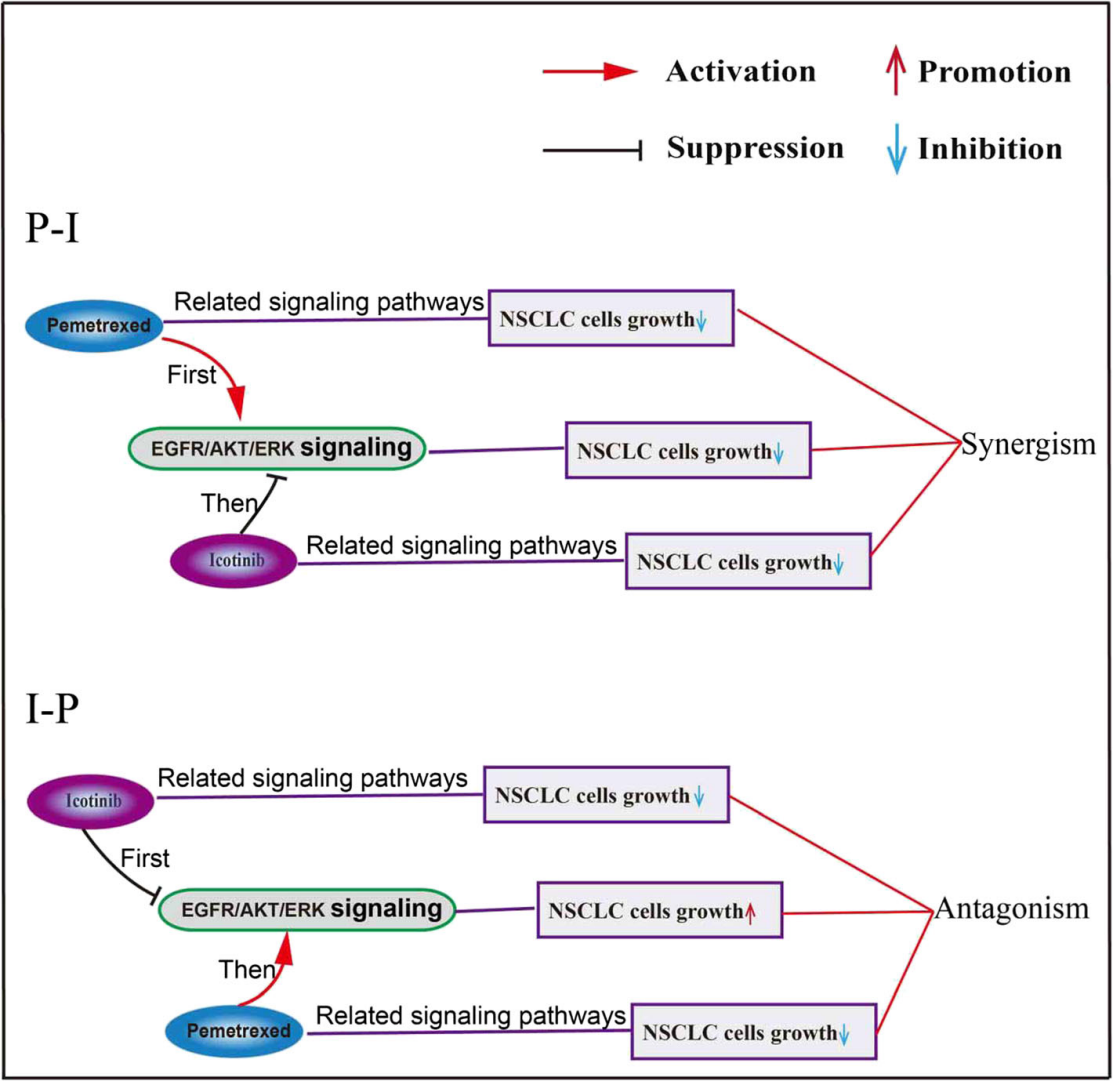
Schematic model: Sequence-dependent synergistic cytotoxicity of icotinib and pemetrexed in human lung cancer cell lines

In this study, we evaluated the response of non-small cell lung cancer (NSCLC) cells PC-9, H1975 and A549 to icotinib and pemetrexed in different orders. We found that with different orders, icotinib enhanced the inhibition effects of pemetrexed on cell growth, colony formation, cell migration, cell invasion, and apoptosis. Our results showed that the sequence of pemetrexed followed by icotinib led to a synergistic effect. At last, we found the synergistic effect of pemetrexed followed by icotinib is mediated through modulation of the EGFR/ERK/AKT, cyclinA/E/p27 and cytochrome-C/Caspase/Bcl-2 signaling pathways (Fig. 9).

EGFR signal transduction pathways play an instrumental role in several cellular processes, such as growth, differentiation, metastasis, and angiogenesis. Two important downstream signaling pathways of EGFR are Raf/MEK/ERK and PI3K/AKT, which are involve in cell proliferation and anti-apoptosis [24–26]. Many studies have showed that compared with standard chemotherapy, EGFR tyrosine kinase inhibitors (EGFR-TKIs) such as gefitinib or erlotinib significantly improve progression-free survival (PFS) in NSCLC patients with EGFR mutated by targeting EGFR signaling pathways. In our study, icotinib down-regulated the expression of p-EGFR, p-ERK, and p-AKT, leading to supression of both the cell survival pathway and cell anti-apoptosis pathway mentioned above. Some studies also demonstrated that cytotoxic agents such as paclitaxel not only activated EGFR signal transduction pathways that led to apoptosis, but also activated EGFR and its downstream signaling pathway [27–30]. In the present study, we also found the levels of p-EGFR and p-ERK increased significantly when cells were exposed to pemetrexed, which means pemetrexed can also activates cell survival pathways. In addition, pemetrexed can also up-regulated the expression of p-AKT whose activation is linked to apoptosis inhibition. Therefore, our result proved pemetrexed can activate both cell survival pathways and cell anti-apoptosis pathways. This may be the reason why many cytotoxic drugs can not eliminate tumor completely. Hence, to some extent, icotinib plays an opposite role in EGFR signal pathways compared with pemetrexed. When combined together, we found icotinib treatment after pemetrexed blocked the survival and anti-apoptosis pathways activated initially by pemetrexed, which contributed to synergetic antiproliferative activity. These findings are consistent with the prior studies that mitogen-activated protein kinase inhibition can selectively enhance the paclitaxel(taxol)-induced cell death in human cancer cell lines [31]. Another study with similar result showed that AKT inhibition synergises with EGFR TKI to increase cell killing in EGFR mutated NSCLC cells [32]. Though some studies indicated that the overall survival in the patients receiving EGFR-TKIs and chemotherapy together was significant longer than that in patients receiving chemotherapy or TKI alone. Unfortunately, four previous studies (INTACT-1, INTACT-2, TALENT and TRIBUTE) showed that directly combined standard platinum-based regimens with gefitinib or erlotinib ended up in failure [12–15]. The explanation for it may be the antagonistic effect generated between EGFR-TKIs and chemotherapeutics. In our study, antagonism was also found when cell lines were exposed to icotinib followed by pemetrexed, which is in agreement with the observations of other four studies [33–36]. When it comes to the mechanism of antagonism, our results showed pemetrexed reactivated the survival and anti-apoptosis pathways blocked by pretreatment with icotinib.

Apoptosis plays a crucial role in the response of cancer to chemotherapy and radiation therapy. Our results showed icotinib could enhance pemetrexed’s pro-apoptosis effect markedly when administered after pemetrexed while icotinib alone could not induce apoptosis. We further found icotinib enhanced pemetrexed’s pro-apoptosis effect via cytochrome-C/Caspase/Bcl-2 signaling pathway.

As many antitumor drugs are cell cycle specific agents, and cell cycle is important to cell proliferation and tumor growth, it is necessary to investigate the change of cell cycle distribution after treatment of icotinib and pemetrexed. A possible explanation for the antagonism generated with sequence of icotinib followed by pemetrexed is that icotinib arrested cells in G1 phase while pemetrexed blocked cells in S phase, and the G1-phase arrest of tumor cells by icotinib protected tumor cells from the S phase-specific pemetrexed. Regarding to the concomitant treatment with pemetrexed and icotinib that resulted in different combined effect in three cell lines, it might be caused by the different time length required by the drugs to transuct signals in different cell lines, but the exact mechanism needs further study.

The mechanism of how pemetrexed, a cytotoxic drug, activates EGFR and its downstream signal pathway remains unknown. Previous studies suggested the following potential mechanisms [37–40]: 1) Cytotoxic drugs activate EGFR and its downstream signal pathway by stimulating and releasing TGFα (a ligand that binds EGFR); 2) Cytotoxic drugs degrade cdc25A phosphatase which may directly modulate phosphorylation of EGFR. Icotinib, however, has explicit mechanism on inactivating EGFR and its downstream signal pathway. It specifically and competitively binds to the ATP-binding site at different levels in EGFR kinase domain, sequentially inhibits kinase activity and thus blocks relevant signal conduction of proliferation and metastasis [19]. Interestingly, icotinib alone could not induce cell apoptosis, consistent with the result of Gao et al. [40], but icotinib was able to significantly enhance the pro-apoptotic effect of pemetrexed, suggesting that icotinib could not initiate cell apoptosis, but might act on downstream signal molecules of apoptosis to strengthen the pro-apoptotic effect of pemetrexed.

In the study of FAST-ACT [41], the sequential cooperativity regimen was implemented by first using cytotoxic drug on day 1 and 8, and EGFR-TKI from day 15 to 28. Hence, the time from the termination of the previous treatment course to the start of the next course is about 2~3 days, which we call washout period. The washout period is very important because the residual EGFR-TKIs can generate antagonism to chemotherapeutics from next course. Our result showed that it took no less than 96 h to completely clear the effect of icotinib on the cells, implicating that chemotherapy should be administered after discontinuation of EGFR-TKIs for at least 96 h so as to prevent antagonistic effect. In fact, due to the different environment between in vitro cell culture and in vivo cell growth as well as drug clearance mechanism, the duration of washout period in the clinical application should be determined by clinical trial.

FASTACT-2 [42] was a large, randomized, controlled phase III clinical trial and showed that administering gemcitabine before erlotinib improved the overall survival only in patients with EGFR mutation. Our study demonstrated that regardless of the mutation status of EGFR, administering pemetrexed before icotinib led to synergistic effect in three cell lines. The reason for it requires further research.

## Conclusion

Our study leads to a conclusion that the synergistic effect of pemetrexed and icotinib in NSCLC cell lines is related to the administration sequence instead of EGFR mutations. The model of pemetrexed followed by icotinib is superior to icotinib followed by pemetrexed or concomitant administration. The mechanism is achieved by the opposite effects of pemetrexed and icotinib on EGFR and its downstream signaling pathway. Therefore, the detection of EGFR and its downstream moleculars phosphorylation levels after pemetrexed treatment may help to predict the efficacy of icotinib in treatment and design rational combination therapies for patients with NSCLC.

## Abbreviations

CytoC: Cytochrome c
EGFR-TKIs: Epidermal growth factor receptor tyrosine kinase inhibitors
I: Icotinib
IF: Immunofluorescence
NSCLC: Non-small cell lung cancer
P: Pemetrexed

## References

[1] Cao S, Wang Z, Gao X, He W, Cai Y, Chen H, Xu R (2018). FOXC1 induces cancer stem cell-like properties through upregulation of beta-catenin in NSCLC. J Exp Clin Cancer Res.

[2] Hoffman PC, Mauer AM, Vokes EE (2000). Lung cancer. Lancet.

[3] Yang C, Wang H, Zhang B, Chen Y, Zhang Y, Sun X, Xiao G, Nan K, Ren H, Qin S (2016). LCL161 increases paclitaxel-induced apoptosis by degrading cIAP1 and cIAP2 in NSCLC. J Exp Clin Cancer Res.

[4] Fisher MD, D'Orazio A (2000). Phase ii and iii trials: comparison of four chemotherapy regimens in advanced non small-cell lung cancer (ecog 1594). Clin Lung Cancer.

[5] Schiller JH, Harrington D, Belani CP, Langer C, Sandler A, Krook J, Zhu J, Johnson DH (2002). Comparison of four chemotherapy regimens for advanced non-small-cell lung cancer. N Engl J Med.

[6] Shepherd FA, Rodrigues Pereira J, Ciuleanu T, Tan EH, Hirsh V, Thongprasert S, Campos D, Maoleekoonpiroj S, Smylie M, Martins R, van Kooten M, Dediu M, Findlay B, Tu D, Johnston D, Bezjak A, Clark G, Santabarbara P, Seymour L (2005). Erlotinib in previously treated non-small-cell lung cancer. N Engl J Med.

[7] Mok TS, Wu YL, Thongprasert S, Yang CH, Chu DT, Saijo N, Sunpaweravong P, Han B, Margono B, Ichinose Y, Nishiwaki Y, Ohe Y, Yang JJ, Chewaskulyong B, Jiang H, Duffield EL, Watkins CL, Armour AA, Fukuoka M (2009). Gefitinib or carboplatin-paclitaxel in pulmonary adenocarcinoma. N Engl J Med.

[8] Mitsudomi T, Morita S, Yatabe Y, Negoro S, Okamoto I, Tsurutani J, Seto T, Satouchi M, Tada H, Hirashima T, Asami K, Katakami N, Takada M, Yoshioka H, Shibata K, Kudoh S, Shimizu E, Saito H, Toyooka S, Nakagawa K, Fukuoka M (2010). Gefitinib versus cisplatin plus docetaxel in patients with non-small-cell lung cancer harbouring mutations of the epidermal growth factor receptor (wjtog3405): An open label, randomised phase 3 trial. Lancet Oncol.

[9] Rosell R, Carcereny E, Gervais R, Vergnenegre A, Massuti B, Felip E, Palmero R, Garcia-Gomez R, Pallares C, Sanchez JM, Porta R, Cobo M, Garrido P, Longo F, Moran T, Insa A, De Marinis F, Corre R, Bover I, Illiano A, Dansin E, de Castro J, Milella M, Reguart N, Altavilla G, Jimenez U, Provencio M, Moreno MA, Terrasa J, Munoz-Langa J, Valdivia J, Isla D, Domine M, Molinier O, Mazieres J, Baize N, Garcia-Campelo R, Robinet G, Rodriguez-Abreu D, Lopez-Vivanco G, Gebbia V, Ferrera-Delgado L, Bombaron P, Bernabe R, Bearz A, Artal A, Cortesi E, Rolfo C, Sanchez-Ronco M, Drozdowskyj A, Queralt C, de Aguirre I, Ramirez JL, Sanchez JJ, Molina MA, Taron M, Paz-Ares L (2012). Erlotinib versus standard chemotherapy as first-line treatment for european patients with advanced egfr mutation-positive non-small-cell lung cancer (eurtac): A multicentre, open-label, randomised phase 3 trial. Lancet Oncol.

[10] Hanna N, Shepherd FA, Fossella FV, Pereira JR, De Marinis F, von Pawel J, Gatzemeier U, Tsao TC, Pless M, Muller T, Lim HL, Desch C, Szondy K, Gervais R, Shaharyar MC, Paul S, Paoletti P, Einhorn L, Bunn PA (2004). Randomized phase iii trial of pemetrexed versus docetaxel in patients with non-small-cell lung cancer previously treated with chemotherapy. J Clin Oncol.

[11] Giaccone G, Herbst RS, Manegold C, Scagliotti G, Rosell R, Miller V, Natale RB, Schiller JH, Von Pawel J, Pluzanska A, Gatzemeier U, Grous J, Ochs JS, Averbuch SD, Wolf MK, Rennie P, Fandi A, Johnson DH (2004). Gefitinib in combination with gemcitabine and cisplatin in advanced non-small-cell lung cancer: a phase iii trial--intact 1. J Clin Oncol.

[12] Herbst RS, Giaccone G, Schiller JH, Natale RB, Miller V, Manegold C, Scagliotti G, Rosell R, Oliff I, Reeves JA, Wolf MK, Krebs AD, Averbuch SD, Ochs JS, Grous J, Fandi A, Johnson DH (2004). Gefitinib in combination with paclitaxel and carboplatin in advanced non-small-cell lung cancer: a phase iii trial--intact 2. J Clin Oncol.

[13] Gatzemeier U, Pluzanska A, Szczesna A, Kaukel E, Roubec J, De Rosa F, Milanowski J, Karnicka-Mlodkowski H, Pesek M, Serwatowski P, Ramlau R, Janaskova T, Vansteenkiste J, Strausz J, Manikhas GM, Von Pawel J (2007). Phase iii study of erlotinib in combination with cisplatin and gemcitabine in advanced non-small-cell lung cancer: the tarceva lung cancer investigation trial. J Clin Oncol.

[14] Herbst RS, Prager D, Hermann R, Fehrenbacher L, Johnson BE, Sandler A, Kris MG, Tran HT, Klein P, Li X, Ramies D, Johnson DH, Miller VA (2005). Tribute: a phase iii trial of erlotinib hydrochloride (osi-774) combined with carboplatin and paclitaxel chemotherapy in advanced non-small-cell lung cancer. J Clin Oncol.

[15] Weeks LD, Zentner GE, Scacheri PC, Gerson SL (2014). Uracil DNA glycosylase (ung) loss enhances DNA double strand break formation in human cancer cells exposed to pemetrexed. Cell Death Dis.

[16] Gridelli C, Kaukel E, Gregorc V, Migliorino MR, Muller TR, Manegold C, Favaretto A, Martoni A, Caffo O, Schmittel A, Rossi A, Russo F, Peterson P, Munoz M, Reck M (2007). Single-agent pemetrexed or sequential pemetrexed/gemcitabine as front-line treatment of advanced non-small cell lung cancer in elderly patients or patients ineligible for platinum-based chemotherapy: a multicenter, randomized, phase ii trial. J Thorac Oncol.

[17] Scagliotti GV, Parikh P, von Pawel J, Biesma B, Vansteenkiste J, Manegold C, Serwatowski P, Gatzemeier U, Digumarti R, Zukin M, Lee JS, Mellemgaard A, Park K, Patil S, Rolski J, Goksel T, de Marinis F, Simms L, Sugarman KP, Gandara D (2008). Phase iii study comparing cisplatin plus gemcitabine with cisplatin plus pemetrexed in chemotherapy-naive patients with advanced-stage non-small-cell lung cancer. J Clin Oncol.

[18] Ciuleanu T, Brodowicz T, Zielinski C, Kim JH, Krzakowski M, Laack E, Wu YL, Bover I, Begbie S, Tzekova V, Cucevic B, Pereira JR, Yang SH, Madhavan J, Sugarman KP, Peterson P, John WJ, Krejcy K, Belani CP (2009). Maintenance pemetrexed plus best supportive care versus placebo plus best supportive care for non-small-cell lung cancer: a randomised, double-blind, phase 3 study. Lancet.

[19] Zhao Q, Shentu J, Xu N, Zhou J, Yang G, Yao Y, Tan F, Liu D, Wang Y, Zhou J (2011). Phase i study of icotinib hydrochloride (bpi-2009h), an oral egfr tyrosine kinase inhibitor, in patients with advanced nsclc and other solid tumors. Lung cancer (Amsterdam, Netherlands).

[20] Tan F, Shen X, Wang D, Xie G, Zhang X, Ding L, Hu Y, He W, Wang Y, Wang Y (2012). Icotinib (bpi-2009h), a novel egfr tyrosine kinase inhibitor, displays potent efficacy in preclinical studies. Lung Cancer.

[21] Shi Y, Zhang L, Liu X, Zhou C, Zhang L, Zhang S, Wang D, Li Q, Qin S, Hu C, Zhang Y, Chen J, Cheng Y, Feng J, Zhang H, Song Y, Wu YL, Xu N, Zhou J, Luo R, Bai C, Jin Y, Liu W, Wei Z, Tan F, Wang Y, Ding L, Dai H, Jiao S, Wang J, Liang L, Zhang W, Sun Y (2013). Icotinib versus gefitinib in previously treated advanced non-small-cell lung cancer (icogen): a randomised, double-blind phase 3 non-inferiority trial. Lancet Oncol.

[22] Brunelle JK, Zhang B (2010). Apoptosis assays for quantifying the bioactivity of anticancer drug products. Drug Resist Updat.

[23] Zhang W, Xu J, Ji D, Li Z, He W, Yang F, Lan H, Wang Y, Wu Z, Liu X, Huang S, Li L, Zhou W (2017). Cycling1 amplification enhances aurora kinase inhibitor-induced polyploid resistance and inhibition of bcl-2 pathway reverses the resistance. Cell Physiol Biochem.

[24] Cheng HW, Chen YF, Wong JM, Weng CW, Chen HY, Yu SL, Chen HW, Yuan A, Chen JJ (2017). Cancer cells increase endothelial cell tube formation and survival by activating the pi3k/akt signalling pathway. J Exp Clin Cancer Res.

[25] Li YC, He SM, He ZX, Li M, Yang Y, Pang JX, Zhang X, Chow K, Zhou Q, Duan W, Zhou ZW, Yang T, Huang GH, Liu A, Qiu JX, Liu JP, Zhou SF (2014). Plumbagin induces apoptotic and autophagic cell death through inhibition of the pi3k/akt/mtor pathway in human non-small cell lung cancer cells. Cancer Lett.

[26] MacKeigan JP, Taxman DJ, Hunter D, Earp HS, Graves LM, Ting JP (2002). Inactivation of the antiapoptotic phosphatidylinositol 3-kinase-akt pathway by the combined treatment of taxol and mitogen-activated protein kinase kinase inhibition. Clin Cancer Res.

[27] Blagosklonny MV, Schulte T, Nguyen P, Trepel J, Neckers LM (1996). Taxol-induced apoptosis and phosphorylation of bcl-2 protein involves c-raf-1 and represents a novel c-raf-1 signal transduction pathway. Cancer Res.

[28] Blagosklonny MV, Giannakakou P, el-Deiry WS, Kingston DG, Higgs PI, Neckers L, Fojo T (1997). Raf-1/bcl-2 phosphorylation: a step from microtubule damage to cell death. Cancer Res.

[29] Torres K, Horwitz SB (1998). Mechanisms of taxol-induced cell death are concentration dependent. Cancer Res.

[30] McDaid HM, Horwitz SB (2001). Selective potentiation of paclitaxel (taxol)-induced cell death by mitogen-activated protein kinase kinase inhibition in human cancer cell lines. Mol Pharmacol.

[31] Bokobza SM, Jiang Y, Weber AM, Devery AM, Ryan AJ (2014). Combining akt inhibition with chloroquine and gefitinib prevents compensatory autophagy and induces cell death in egfr mutated nsclc cells. Oncotarget.

[32] Cheng H, An SJ, Zhang XC, Dong S, Zhang YF, Chen ZH, Chen HJ, Guo AL, Lin QX, Wu YL (2011). In vitro sequence-dependent synergism between paclitaxel and gefitinib in human lung cancer cell lines. Cancer Chemother Pharmacol.

[33] Li T, Lara PN, Mack PC, Perez-Soler R, Gandara DR (2010). Intercalation of erlotinib and pemetrexed in the treatment of non-small cell lung cancer. Curr Drug Targets.

[34] Li T, Ling YH, Goldman ID, Perez-Soler R (2007). Schedule-dependent cytotoxic synergism of pemetrexed and erlotinib in human non-small cell lung cancer cells. Clin Cancer Res.

[35] Chen B, Zheng J, Zeng Y, Li B, Xie B, Zheng J, Zhou J, Zhang W (2014). Sequence-dependent antiproliferative effects of gefitinib and docetaxel on non-small cell lung cancer (nsclc) cells and the possible mechanism. PLoS One.

[36] Cheng H, An SJ, Dong S, Zhang YF, Zhang XC, Chen ZH, Jian S, Wu YL (2011). Molecular mechanism of the schedule-dependent synergistic interaction in egfr-mutant non-small cell lung cancer cell lines treated with paclitaxel and gefitinib. J Hematol Oncol.

[37] Morgan MA, Parsels LA, Parsels JD, Mesiwala AK, Maybaum J, Lawrence TS (2005). Role of checkpoint kinase 1 in preventing premature mitosis in response to gemcitabine. Cancer Res.

[38] Wang Z, Zhang B, Wang M, Carr BI (2005). Cdc25a and erk interaction. Egfr-independent erk activation by a protein phosphatase cdc25a inhibitor, compound 5. J Cell Physiol.

[39] Furet P, Caravatti G, Lydon N, Priestle JP, Sowadski JM, Trinks U, Traxler P (1995). Modelling study of protein kinase inhibitors: binding mode of staurosporine and origin of the selectivity of cgp 52411. J Comput Aided Mol Des.

[40] Gao Z, Chen W, Zhang X, Cai P, Fang X, Xu Q, Sun Y, Gu Y (2013). Icotinib, a potent and specific egfr tyrosine kinase inhibitor, inhibits growth of squamous cell carcinoma cell line a431 through negatively regulating akt signaling. Biomed Pharmacother.

[41] Mok TS, Wu YL, Yu CJ, Zhou C, Chen YM, Zhang L, Ignacio J, Liao M, Srimuninnimit V, Boyer MJ, Chua-Tan M, Sriuranpong V, Sudoyo AW, Jin K, Johnston M, Chui W, Lee JS (2009). Randomized, placebo-controlled, phase ii study of sequential erlotinib and chemotherapy as first-line treatment for advanced non-small-cell lung cancer. J Clin Oncol.

[42] Wu YL, Lee JS, Thongprasert S, Yu CJ, Zhang L, Ladrera G, Srimuninnimit V, Sriuranpong V, Sandoval-Tan J, Zhu Y, Liao M, Zhou C, Pan H, Lee V, Chen YM, Sun Y, Margono B, Fuerte F, Chang GC, Seetalarom K, Wang J, Cheng A, Syahruddin E, Qian X, Ho J, Kurnianda J, Liu HE, Jin K, Truman M, Bara I, Mok T (2013). Intercalated combination of chemotherapy and erlotinib for patients with advanced stage non-small-cell lung cancer (fastact-2): a randomised, double-blind trial. Lancet Oncol.

